# PET Fibers Modified with Cloisite Nanoclay

**DOI:** 10.3390/polym12040774

**Published:** 2020-04-01

**Authors:** Janusz Fabia, Andrzej Gawłowski, Monika Rom, Czesław Ślusarczyk, Anna Brzozowska-Stanuch, Marta Sieradzka

**Affiliations:** 1 Institute of Textile Engineering and Polymer Materials, University of Bielsko-Biala, Willowa 2, 43-309 Bielsko-Biala, Poland; agawlowski@ath.bielsko.pl (A.G.); cslusarczyk@ath.bielsko.pl (C.Ś.); msieradzka@ath.bielsko.pl (M.S.); 2 BOSMAL Automotive Research and Development Institute Ltd., Sarni Stok 93,43-300 Bielsko-Biala, Poland; Anna.Brzozowska-Stanuch@bosmal.com.pl

**Keywords:** PET fibers, Cloisite, flame retardancy, LOI, TGA, FTIR, SEM, WAXS, SAXS

## Abstract

The alternative method of reducing the flammability of polyethylene terephthalate (PET) fibers, analogous to dyeing of PET fibers with dispersed dyes in a high-temperature bath, was proposed. A commercial organophilic montmorillonite Cloisite^®^15A (C15A) was applied as a flame retardant. The aim of the presented work was to evaluate the effectiveness of the introduced modifier and the improvement of the flame-retardant properties of PET fibers by limiting oxygen index (LOI) and thermogravimetric analysis (TGA) measurements. Evolved gas analysis (EGA) by spectrometric method (FTIR) during coupled thermogravimetric analysis (TGA) was applied in order to confirm no increase in the toxicity of volatile degradation products released from burning modified fibers. The nanocomposite nature of modified fibers was confirmed based on the structural parameters of the fibers determined using wide-angle X-ray scattering (WAXS) and small angle X-ray scattering (SAXS) X-ray diffraction methods.

## 1. Introduction

Polyethylene terephthalate (PET) fibers are produced in very large quantities worldwide, due to their relatively low cost. Moreover, the fibers are characterized by very good mechanical properties, high resistance to physical and chemical agents. Therefore, they are very widely used as fibers for textiles, technical products (e.g., cords for tires, safety belts) and the production of articles intended for interior furnishings of public facilities or car upholstery. Due to such a wide range of applications, products from PET fibers are facing very strict requirements in terms of fire safety. Standard PET fibers are not very resistant to fire and large amounts of smoke are emitted during their combustion. In addition, the so-called dripping effect is observed; detached drops of molten polymer burn and promote the spread of fire. Therefore, imparting flame-retardant properties and resistance to dripping to PET fibers is of particular interest to researchers [1]. The flame-retardant modification of PET fibers can be achieved in various ways. Most often, a properly ground (micro- or nano-) anti-pyrene is added to the polymer melt [2,3,4], but also a surface application of the flame retardant is employed during the impregnation process [5,6,7,8,9]. The chemical method is used less often. An example here may be the introduction of the aryl ether monomer (2,2′- (4,4′ - (1,4-phenylenebis (oxy)) bis (4,1-phenylene)) bis (oxy) diethanol—PBPBD) into the PET backbone chain during polycondensation [10].

In recent years, montmorillonite (MMT) nanoclay has been used to modify polymers, thus providing an improvement in performance, including fire resistance. Nanoclays used as an additive to the polymer melt significantly improve the flame-retardant properties of the material, especially if they are used in synergistic systems with other non-halogenated anti-pyrenes.

The addition of Cloisite 15A to the ethylene-vinyl acetate (EVA) melt in combination with phosphorus compounds increases the flame-retardant properties of the tested blend. Observation of the charred residue revealed the formation of a dense and stable layer, which is an effective barrier against the penetration of oxygen and heat and the release of flammable gases [11]. Increased flame-retardant properties can be the result of physical and thermal cross-linking of nano-clay particles and the polymer chain and physicochemical adsorption of volatile degradation products on silicates (depending on the organic modifier of montmorillonite) [12]. 

There are certain technological difficulties in production of fibers from thermoplastic polymers with the addition of nanoclay using extruder method. The example might be the process of spinning and stretching of polypropylene fibers with clay addition above 1% by weight, which is technically unacceptable due to the loss of spinnability of the polymer melt [13]. The dispersion of clay nanoparticles is an important technological parameter when adding clay nanoparticles to the polymer melt. Electron microscopy (TEM) and X-ray diffraction (XRD) methods are particularly useful for assessing dispersion in a polymer matrix [14]. The presence of montmorillonite clay particles in the PET matrix affects the crystallization of the polymer, lowers the glass transition temperature and leads to the heterogeneous nucleation of the composite [15]. Further studies have shown that the addition of the clay causes the crystallization temperature to drop. This means that organophilic montmorillonite promotes PET crystal nucleation and even a small amount of organic clay is sufficient to maximize the nucleation effect [16]. In addition, surface morphology studies using the TEM method showed partial peeling of the surface structure of PET-CLAY nanocomposite film (addition of 3% C30B) [17].

An effective way of increasing flame resistance is also coating of the finished materials with special pastes containing a flame-retardant admixture. However, the resulting coatings change the surface and functional properties of processed products. It has been proven that the use of nanoclay for varnish coatings in optimal amounts of 3–5% significantly improves the flame retardancy of the impregnated product [18]. Addition of nanoclay below 1% and above 10% does not result in the desired effects. The type of nanoclay used is also important, as confirmed by flammability tests. The use of polyurethane resins with the addition of montmorillonite in form of Cloisite 30B as a coating on polyester and cotton fabrics also effectively protects the impregnated material against the harmful effects of fire [19]. The admixture of montmorillonite acts, therefore, as a flammability inhibitor.

The method of applying nanoclay in the form of an aqueous dispersion on cotton fabric was described in [20]. In this way, the limiting oxygen index (LOI) was increased by 4% for nanoclay processing, while for fabric activated by low-temperature plasma in nitrogen and then treated with nanoclay, a 5% increase in oxygen index was achieved. Such an effect of synergistic interactions was similar to the previously discussed citations.

However, there are no reports in the literature regarding the use of an analogous, aqueous dispersion of organophilized aluminosilicate for the preparation of flame-retardant polyester fibers, the possibility of flame-retardant treatment based on the application of natural zeolite was studied [21]. Therefore, in order to fill the existing gap, it was decided to carry out the relevant research, the results of which are described in this study. Commercial Cloisite 15A type montmorillonite, ground to nanometric size, was used for flame-retardant modification of PET fibers. The criterion for choosing this type of clay was the interlayer distance, which was the largest among the entire Cloisite series. The flame-retardant application was carried out using the exhaust method. This method involves the application of fiber treatment in water bath containing modifier at an appropriately high temperature (HT) and is analogous to dyeing using disperse dyes. The proposed method has been successfully tested before in the same, optimized process conditions, with the use of other flame retardants [22,23,24].

As a result of using the proposed method, modified PET fibers were obtained and comprehensively tested. Description of the introduction of C15A clay nanoparticles into the fiber material was based on the so-called voids approach [25]. Three main goals of this study were set: to assess the effectiveness of improving the flame-retardant properties of fibers, to analyze gaseous oxidative degradation products in terms of their toxicity in the event of a fire, and to describe the changes in the supermolecular structure of fibers resulting from the modification.

## 2. Materials and Methods

The materials used in this work were all commercially available technical products. PET fibers were supplied by Elana SA Toruń (Toruń, Poland). As the flame-retardant modifier—organically modified montmorillonite in the form of Cloisite^®^15A nanoclay, produced by Southern Clay Product Ltd. (USA), was used [26]. The finishing treatment of fibers was carried out in the laboratory dyeing device (Ahiba Turbomat—Lucerne, Switzerland) with a liquor ratio of 1:50. The conditions established were the following: temperature—130 °C, treatment time—1 h and heating rate—2.5 °C/min, respectively. The PET fibers were processed in an aqueous dispersion containing the C15A modifier and Rokacet R40 KO300G (Glyceryl Cocoate; CAS no. 68201-46-7) nonionic surfactant supplied by PCC Group (Brzeg Dolny, Poland) which was added into the bath in the amount of 0.7 g/L. C15A nanoclay dispersion was prepared using an ultrasonic bath for 15 min. After the treatment samples were washed in a solution of detergent Pretepon G (PCC Group—Brzeg Dolny, Poland) in the amount of 5 g/L. The washing time was 30 min and the temperature was 60 °C, washing was carried out in the laboratory dyeing device (Ahiba Turbomat—Lucerne, Switzerland). The effectiveness of the modifier was tested in a wide range of flame-retardant concentrations, ranging from 0 to 7.5% in relation to the fiber weight.

The examinations of the fiber flammability were carried out using the limiting oxygen index (LOI) method in accordance with PN ISO 4589.

Thermogravimetric analysis (TGA) was performed using Thermogravimetric Analyzer Q500 TA Instruments (New Castle, DE, USA). Measurements were carried out in a temperature range from 30 to 700 °C with the heating rate of 20 °/min in the air atmosphere (flow 60 mL/min). Pre-tared platinum pans were used to contain the samples, and the mass of the sample was between 20 and 30 mg. The data were evaluated by means of the Universal V4.5A (TA Instruments, (New Castle, DE, USA) software.

Evolved gas analysis (EGA) by spectrometric method (FTIR) during coupled thermogravimetric analysis (TGA) measurements were carried out using TA Instruments Q500 thermobalance (New Castle, DE, USA) with a special EGA furnace coupled with the Nicolet iS50 FTIR spectrometer (Thermo Fisher Scientific, Waltham, MA, USA). The furnace incorporates a quartz liner between the bifilar-wound heating element and the sample measurement area. This arrangement results in a small internal volume that is readily swept at normal purge rates, assuring rapid transfer and reduced dilution of decomposition off-gases. Test parameters were the following: platinum crucible, heating rate: 20 °C/min, temperature range: 30.0–750.0 °C, gas flow: air 50 mL/min. The gas flow rate through the transfer line and gas cell was kept constant. Infrared spectra over the range of 4000 to 450 cm^−1^ were collected every 15 s at a resolution of 4 cm^−1^. The absorption bands of each recorded spectrum were simultaneously integrated over the entire spectral range. Gram-Schmidt (G-S) curves were obtained by plotting integration from each spectrum as a function of time. The average intensity of volatiles during the mass loss was determined and depicted in the G-S curves based on vector analysis. FTIR spectra analysis was performed using OMNIC Series 9.8.372 software (Thermo Fisher Scientific, Waltham, MA, USA) and a Nicolet™ FTIR Vapor Phase Spectral Library (Thermo Fisher Scientific, Waltham, MA, USA).

The wide-angle X-ray scattering (WAXS) investigations were carried out with a URD-65 Seifert (Rich. Seifert & Co. Röntgenwertk, Ahrensburg, Germany) diffractometer. CuKα radiation was used at 40 kV and 30 mA. Monochromatization of the beam was obtained by means of a nickel filter and a graphite crystal monochromator placed in the diffracted beam path. A scintillation counter was used as a detector. Investigations were performed in the range of angles from 3° to 40° in steps of 0.1°.

The small angle X-ray scattering (SAXS) experiments were performed by means of an MBraun camera, which utilizes the conventional Kratky collimation system. The front of the camera was directly mounted on the top of the tube shield of a stabilized Philips PW 1830 X-ray generator. The X-ray tube was operated at a power of 1.5 kW. Cu*K*_α_ radiation was used. Scattered radiation was recorded in an acquisition time of 1200 s by means of an MBraun linear position-sensitive detector, model PSD 50 (HECUS-MBraun Graz X-Ray Systems, Graz, Austria). The detector had 1024 channels with a channel-to-channel distance of 52 μm.

Scanning electron microscopy (SEM) analyses were performed in conventional SEM mode using Jeol JSM 5500LV instrument (JEOL Ltd., Tokyo, Japan) operating at 10 kV, after coating the samples with a thin layer of gold by sputter deposition. Surfaces of samples and scorching after the process of burning of PET fibers were observed at different magnifications.

## 3. Results and Discussion

The presentation of the research results is divided into 3 parts corresponding to the main objectives set by the authors: assessment of the effectiveness of the introduction of flame retardant into fiber structure, analysis of gaseous products of the oxidative degradation of fibers and the characterization of the supermolecular structure of the fibers modified with Cloisite C15A.

### 3.1. Studies on the Effectiveness of the Flame-Retardant Modification

The assessment of the effectiveness of the proposed flame-retardant modification includes the determination of the limiting oxygen index (LOI), thermogravimetric tests carried out to confirm the results of LOI tests and microscopic observations of the charred solids obtained as a result of the controlled fiber burning. 

#### 3.1.1. Limiting Oxygen Index

The resulting flame-retardant effect of PET fibers modification was evaluated using the limiting oxygen index (LOI) method. A parameter that characterizes the method and is the lowest percentage of oxygen in the mixture with nitrogen at which the test specimen ignites and burns on its own. The measurements were performed in accordance with PN–ISO 4589 standard. The obtained results are presented in Table 1.

Based on the analysis of the LOI values given in Table 1, it can be concluded that the addition of C15A clay in the nanopowder form to PET fibers indeed changes the value of the limiting oxygen index. The differences in values are not large, but they are undoubtedly significant. The highest LOI value was obtained for PET fibers modified in a bath containing 0.5% C15A in relation to the fiber weight. It should be noted that a further increase in the modifier content results in a systematic reduction of LOI value. The addition of C15A in quantities of above 2.5% causes the oxygen index to decrease to the value lower than when burning PET fibers without a modifier.

#### 3.1.2. Thermogravimetric Analysis

**Figure 1:**
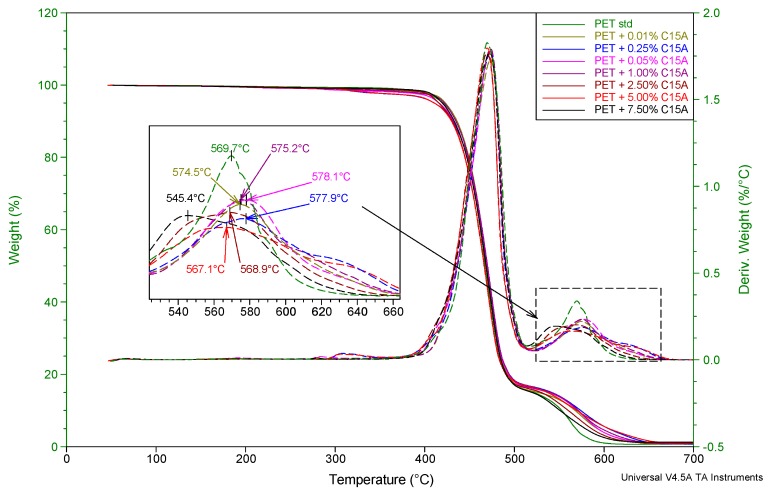
TG and DTG curves of polyethylene terephthalate (PET) fibers modified with Cloisite^®^15A in the bath at 130 °C. Heating rate 20 °/min, purge air flow 60 mL/min.

Thermogravimetric analyses in the air were performed in order to confirm the improvement of the flame-retardant properties of the fibers, represented by the LOI values. In the case of polymers such as PET, thermal dissociation in an oxidizing atmosphere and direct combustion of residues after thermal dissociation are almost fully separable transformations, so that the application of the TGA method gives very good results. Such an approach has already been demonstrated both for classic PET fibers [22] and for partially oriented PET fibers (POY) formed at high speeds [27]. Based on the analysis of the DTG curves, it is possible to show the shift of the exothermic peak (and its maximum) corresponding to the combustion process of the sample. When the material is modified with antipyrene, the peak shift towards higher temperatures is observed. In the observed case, the maximum temperature of the discussed effect (Figure 1) shifts from 569.7 °C for pure PET to 578.1 °C for the most favorable modification variant, that is 0.5% of C15A.

Along with the further increase of the modifier content, which was tested up to 7.5% of C15A, the opposite tendency was observed, i.e., the shift of the discussed maximum towards lower temperatures. The obtained results clearly substantiate the results of the LOI tests and indicate a real improvement of the flame retardancy of the tested PET fibers.

#### 3.1.3. Microscopic Observations of Charred Residues.

**Figure 2:**
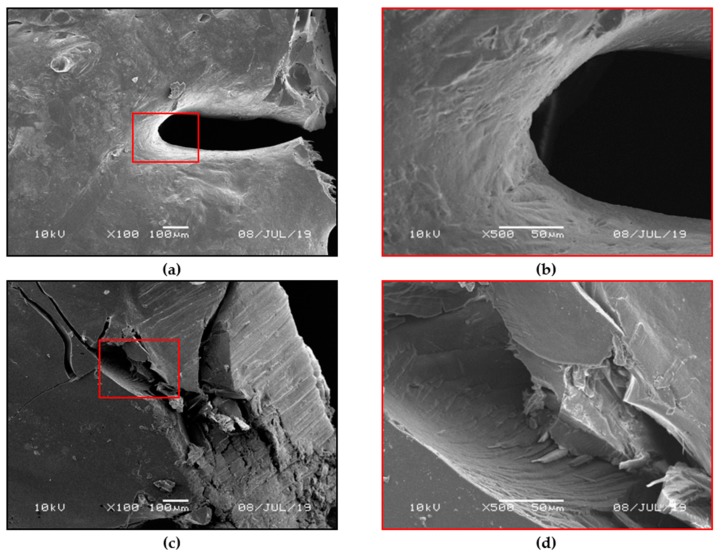
SEM microphotographs of residue after combustion of PET fibers modified with Cloisite^®^15A (C15A) (0.5%; 130 °C) (**a**,**b**). SEM microphotographs of residue after combustion of a sample of unmodified PET fibers (**c**,**d**).

For the optimal variant of fibers after the flame-retardant modification (0.5% C15A addition), determined on the basis of LOI and TGA tests, the controlled combustion of the sample in atmospheric air was carried out, accompanied with the analysis of the burning process and microscopic observation of the charred residues. It was found that during the burning of nanoclay-modified fibers no characteristic phenomenon of dripping was observed. Dripping of molten, burning material, which occurs in the case of raw PET can drastically contribute to the propagation of the flame zone during a fire. SEM microscopic observations of charred residues of nanoclay-modified fibers after combustion (Figure 2a,b) reveal a very clear difference with respect to the material that was not flame retardant (Figure 2c,d).

The surface of the charred residue for the sample with nanoclay is covered with a characteristic coating. The charred residues are formed in the immediate vicinity of the flame so that the said coating effectively limits the access of flammable decomposing gases released during thermal dissociation of PET to the flame zone. Therefore, even after the direct ignition of the material, the tendency to retard fire and limit flame propagation was observed.

The observed phenomenon is consistent with the conclusions of many authors investigating the effect of flame retardancy after the introduction of modified montmorillonite nano-powder into various polymer matrices: PA6, PP, PP/MA [28,29,30], PS [31]. In our case, C15A clay dispersed in water bath was introduced into the material (matrix) of PET fibers not through physical mixing in the melt, but through penetration of the fiber structure and filling of the so-called voids in conditions of increased pressure and temperature (130 °C) for approx. 1 h.

It turns out that under the abovementioned conditions it is possible to obtain a specific structure of reduced gas permeability, described for the first time by K. Yano et al. [32] for MMT-containing polyimide nanocomposites. Since the authors of this study in the near future intend to propose a separate publication dedicated to X-ray studies of delamination of the C15A modifier and to FTIR temperature studies of the solid residue arising from the oxidative degradation process of PET fibers, the burning process inhibition mechanism of modified PET fibers will not be discussed here.

### 3.2. Analysis of Volatile Products of the Oxidative Degradation of Fibers

Another important issue is the analysis of gaseous products released during the oxidative degradation of the modified fibers during their burning process. The increase in toxicity of gases generated during decomposition of polymers in the event of a fire under the influence of specific anti-pyrenes is an extremely important problem. This phenomenon occurs especially when halogenated compounds are used to obtain flame-retardant properties of polymer materials. Despite the extremely high effectiveness of these anti-pyrenes, they have been almost eliminated from the market over the past several years. There are systematic attempts to replace them with anti-pyrenes with a completely different mechanism of flame inhibition, and the results described in this chapter also follow this trend.

#### 3.2.1. C15A Nanoclay

**Figure 3:**
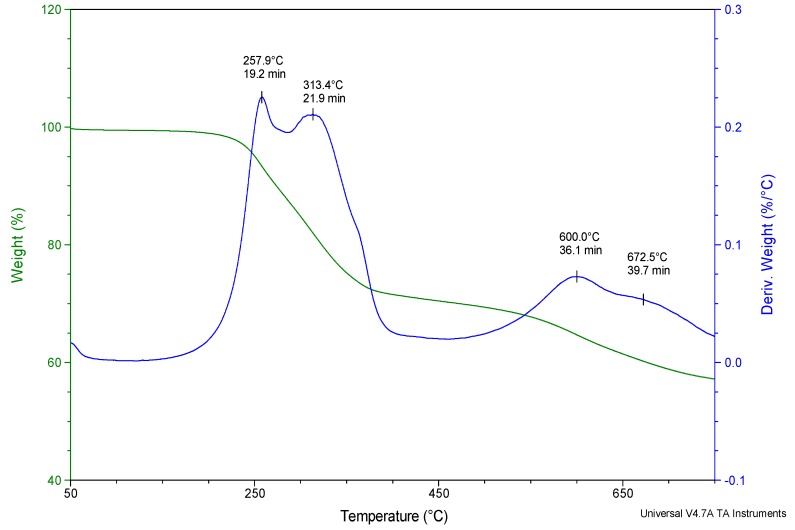
TG and DTG curves of C15A clay with marked FTIR analysis points.

The study of gases emitted during the thermal decomposition of the C15A modifier was carried out based on the analysis of the TG and DTG curves in a wide temperature range from 50 to 750 °C (Figure 3). Local maxima marked on the DTG curve determine the temperatures at which the rate of mass loss associated with the occurrence of specific transformations in the tested material is the highest. These temperatures of 257.9 °C, 313.4 °C, 600.0 °C and 672.5 °C, respectively, served as characteristic temperatures at which FTIR spectra, recorded every 15 s in the whole temperature range, were subjected to a detailed analysis aimed at identifying specific chemical compounds.

**Figure 4:**
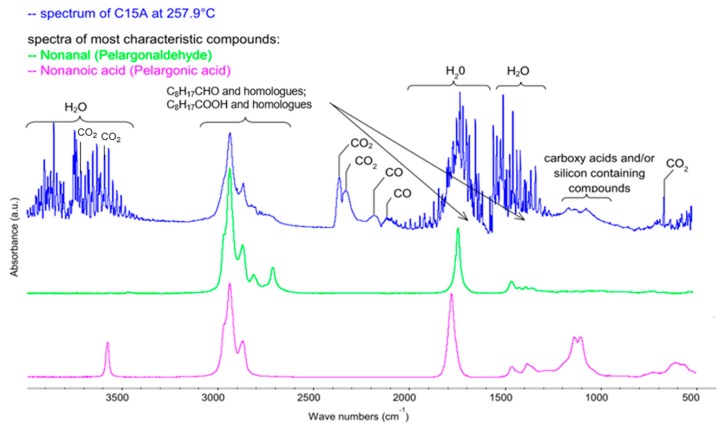
FTIR spectrum of gases evolved during oxidative degradation of C15A recorded at 257.9 °C (blue) with spectra of the most representative compounds from the group of aldehydes and acids—nonanal (green) and nonanoic acid (pink), respectively, present in products at the above temperature. Qualitative characteristics of the spectrum were obtained using Nicolet™ FTIR Vapor Phase Spectral Library software.

The FTIR spectrum recorded at 257.9 °C (Figure 4) illustrates a qualitative mixture of gases released from a C15A nanoclay sample heated in the air, as the effect of the breakdown of the quaternary ammonium salts particles, introduced into the spaces between the silicate layers of aluminosilicate in the organophilization process. In the case of C15A clay, it is a sterically developed compound with 2 characteristic tails, which are essentially simple triacylglycerols containing residues of saturated fatty acids with 18 carbon atoms in the chain [26].

As a result of the thermal dissociation, a series of absorption bands appear in the recorded FTIR spectrum, among others in the ranges of 1350–1450 cm^−1^, 1650–1700 cm^−1^ and 2700–2950 cm^−1^, assigned respectively to the entire group of aldehydes and acids. Using the specialized Nicolet^TM^ FTIR Vapor Phase Spectral Library software, the following most characteristic compounds were identified: C_8_H_17_CHO pelargonic aldehyde (nonanal) and C_8_H_17_COOH pelargonic acid (nonanoic acid). Those products are formed as a result of the breakdown of quaternary ammonium salt used for organophilization of C15A clay, and more precisely the breaking of ester bonds and fragmentation of saturated fatty acid molecules, most often at about half-length of the chain. The appearance of the above compounds in the atmosphere of gases leaving the measurement cell, in the analyzed temperature range, is evidenced by the characteristic smell of geranium. It should be emphasized that these compounds do not present a toxicological hazard.

Pelargonic acid is found in the form of esters in the essential oil obtained from geranium and is a natural non-selective herbicide. In contrast, pelargonium aldehyde (nonanal) is a compound found naturally in some essential oils (including cinnamon and rose oil), and is also produced in small amounts by the human body [33] so that it is not only non-toxic but also biocompatible. In addition to the compounds mentioned above, the discussed FTIR spectrum of C15A nanoclay is predominated by water, carbon dioxide and carbon monoxide.

**Figure 5:**
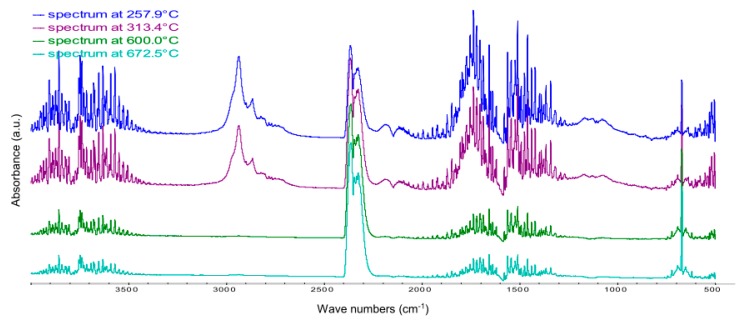
FTIR spectra of gases emitted during oxidative degradation of C15A recorded at: 257.9 °C (19.2 min), 313.4 °C (21.9 min), 600.0 °C (36.1 min) and 672.5 °C (39.7 min).

Analyzing the spectrum recorded at 313.4 °C, i.e., the second maximum on the DTG curve, there are no qualitative changes. The intensity of CO_2_-derived bands increases, while the intensity of absorption bands dedicated to water and carbon monoxide decreases (Figure 5). The intensity of the C_8_H_17_CHO and C_8_H_17_COOH bands and their homologs also decreases. This situation is caused by the fact that the second maximum on the DTG curve corresponds to the same transformations as the ones discussed in the first example (spectrum recorded at 257.9 °C), i.e., the breakdown of the quaternary ammonium salt introduced between silicate stacks and the removal of water from the interlayer spaces of C15A nanoclay. However, this is a two-step process, because the cleavage of ester bonds in triacylglycerols is energetically non-homogeneous, as it depends on which carbon atom in glycerin the given bond is located at.

In the FTIR spectra of the gases emitted as a result of the oxidative degradation of the C15A, recorded at 600.0 °C and 672.5 °C (respectively third and fourth maximum on the DTG curve), only the bands associated with the presence of water and CO_2_ are observed. The intensity of bands dedicated to H_2_O decreases systematically with the increase of temperature, while in the case of bands dedicated to CO_2_ the intensity increases slightly with the temperature (Figure 5).

#### 3.2.2. Pure PET

**Figure 6:**
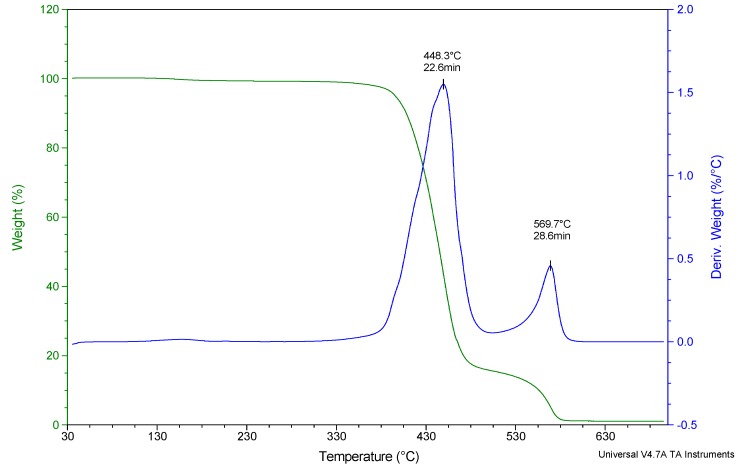
TG and DTG curves of pure PET with marked FTIR analysis points.

Studies on the characterization of gaseous products generated as a result of the oxidative degradation of PET fibers before flame-retardant modification was done in order to settle the reference point for samples after the modification. Selected FTIR spectra were collected at temperatures corresponding to the temperatures of maxima registered on the DTG curve (Figure 6).

**Figure 7:**
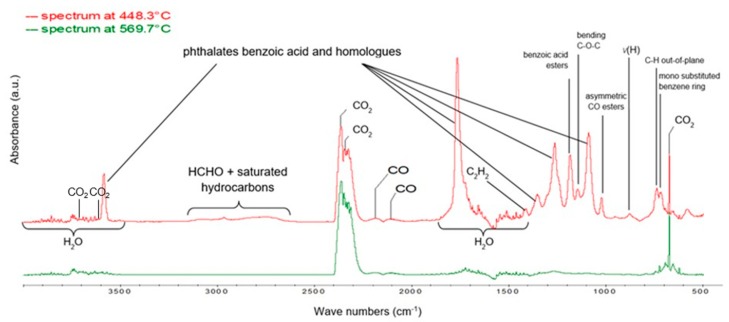
FTIR spectra recorded for pure PET in the range of 500–4000 cm^−1^ at temperatures characteristic of the oxidative degradation process: 448.3 and 569.7 °C, respectively, determined on the basis of DTG curves. Qualitative characteristics of the spectrum were obtained using Nicolet™ FTIR Vapor Phase Spectral Library software and [34].

The first maximum, at 448.3 °C, corresponds to the highest mass loss rate occurring during the thermal dissociation process of the PET fiber material in the oxidizing atmosphere. In the case of pure PET, the recorded FTIR spectrum is characterized by the occurrence of a number of absorption bands corresponding to the main components of volatile oxidative degradation products, including, first of all, pairs of phthalates, benzoic acid and its homologues (main bands, in the range: 1050–1400 cm^−1^, 1780 cm^−1^ and 3600 cm^−1^, respectively), formaldehyde and saturated hydrocarbons (extensive low-intensity band in the range of 2640–3680 cm^−1^), unsaturated hydrocarbons: mainly C_2_H_2_ (1420 cm^−1^) and also characteristic absorption bands of water, carbon dioxide and carbon monoxide (Figure 7).

The FTIR spectrum recorded at 569.7 °C corresponds to the second maximum on the DTG curve. It is associated with the highest mass-loss rate during the combustion of the solid residue after decomposition of polyethylene terephthalate. Among the gases emitted there only CO_2_, small amounts of H_2_O and the traces of CO could be found. Therefore, unlike the gas mixture generated during the oxidative degradation stage of PET, in the case of post-combustion of the char resulting from this degradation, the toxicity of emitted gaseous products is reduced nearly to zero.

#### 3.2.3. PET Modified with C15A

**Figure 8:**
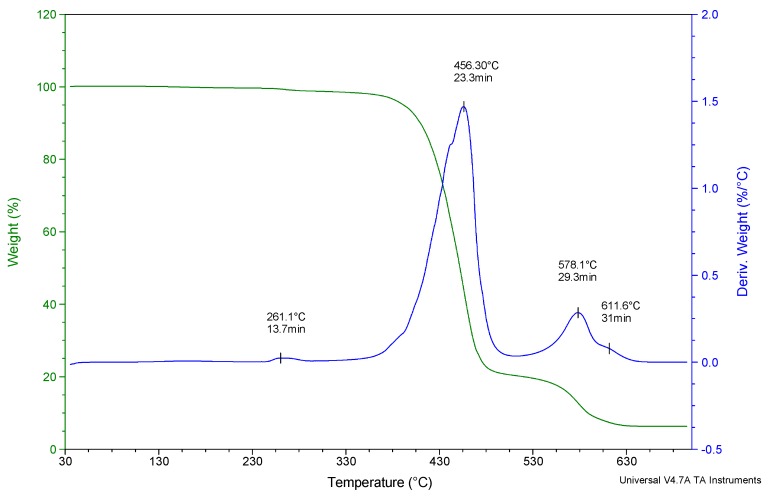
TG and DTG curves of PET modified with C15A (0.5%) with marked FTIR analysis points.

The discussion of the results in this chapter is concluded with the analysis of gaseous products generated during the oxidative degradation of PET fibers subjected to flame-retardant treatment in a bath containing 0.5% of C15A nanoclay (based on fiber weight). For analysis, as in the previously discussed cases, selected FTIR spectra were recorded at the temperatures corresponding to the temperatures of maxima observed on the DTG curves (Figure 8). It is worth noticing that the small local maximum at 261.1 °C corresponds to the cleavage of ester bonds in the molecules of the quaternary ammonium salt present in the interlayer spaces of C15A nanoclay. Despite the relatively low content of the flame retardant used, the change in sample mass associated with the transformation of its chemical structure is reflected in the DTG curve. The beginning of thermal dissociation of C15A is almost perfectly coincident with the temperature range of the physical PET melting process. The temperature of the second maximum on the DTG curve registered for modified fiber, corresponding to the highest mass loss rate resulting from thermal dissociation of polyethylene terephthalate under the oxidative conditions, is higher by ~8 °C as compared to the results obtained for unmodified PET fibers.

Thus, the previously stated theses [31,35] can be confirmed: depending on the type of polymer matrix and montmorillonite content, the temperature of nanocomposites decomposition increased from a few to nearly 50 °C. This phenomenon is explained by the limitation of the mobility of polymer chains located in the interlayer spaces of aluminosilicate. Therefore, it is assumed that in the observed samples exfoliation or at least intercalation of montmorillonite layers occurs. Despite the fact that in the studied case samples were prepared without the physical mixing of C15A nanoclay with polyethylene terephthalate in the melt, but only by exhaustion of MMT from the water bath, i.e., the introduction of modifier particles from the aqueous dispersion, as a result a modified material presenting the properties of nanocomposite was obtained, the structure of which will also be discussed based on the results of X-ray structural analysis.

**Figure 9:**
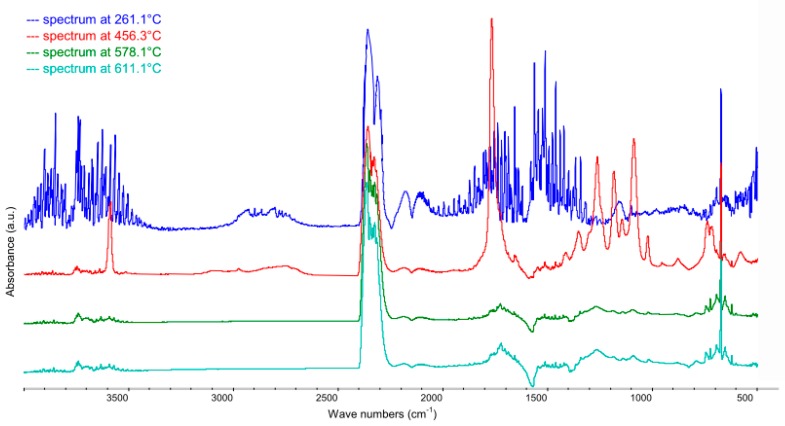
FTIR spectra recorded for PET modified with C15A (0.5%) in the range of 500–4000 cm^−1^ at temperatures characteristic of its oxidative degradation process: 261.1 °C (13.7 min), 456.3 °C (23.3 min), 578.1 °C (29.3 min), 611.1 °C (31.0 min), respectively, determined on the basis of DTG curves.

Analyzing the FTIR spectra recorded for PET fiber + 0.5% of C15A (Figure 9) it should be noted that in addition to the spectrum recorded at 261.1 °C, which is analogous to the spectrum for pure C15A modifier (except for the characteristic bands corresponding to aldehydes and ketones formed after the cleavage of ester bonds, the intensity of which is very clearly reduced in this case), the other spectra are qualitatively identical to those recorded for pure PET fibers.

**Figure 10:**
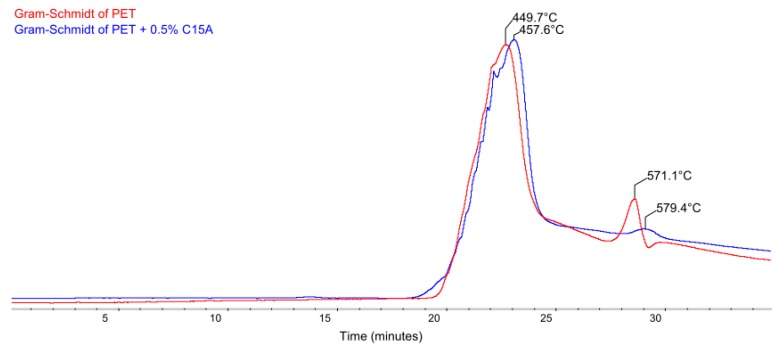
Gram-Schmidt curves of pure PET (red) and PET modified with 0.5% C15A (blue).

In order to enable the quantitative comparison of spectra aimed at determining if the gases emitted as the result of the thermal decomposition of modified fibers are toxic or not, Gram-Schmidt (G-S) curves as a function of time were generated, illustrating the intensity of gaseous product evolution in the entire analyzed temperature range: from 30 to 700 °C (Figure 10).

Each of the G-S curves is based on all the individual spectra. Gram-Schmidt curve is plotted by the calculated values of the infrared extinction coefficient of the evolved gases and their corresponding concentrations over the entire wavenumber range [36]. The peaks appearing on the G-S curves correspond to the maxima on the DTG curves, but are slightly shifted towards higher temperatures. In the presented case, at the heating rate of 20 °C/min, this shift is approx. 1.3–1.4 °C. This shift is a result of the delay between the TGA furnace and the detector in the FTIR. Evolved gases are swept through a transfer line which is kept at 250 °C to avoid condensation of relatively higher molecular weight gaseous products.

**Figure 11:**
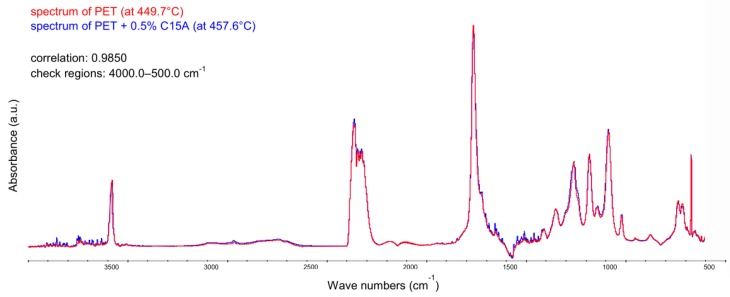
FTIR spectra recorded for pure PET (red) and PET modified with 0.5% C15A (blue) in the range of 500–4000 cm^−1^ at temperatures characteristic of the first step of oxidative degradation process: 449.7 and 457.6 °C, respectively, determined on the basis of Gram-Schmidt curves.

Since toxic gaseous products appear almost exclusively during the decomposition of the base polymer, i.e., PET, it was relevant to compare the spectra recorded at temperatures corresponding to the first maximum on the G-S curves, for pure PET (449.7 °C) and for modified PET + 0.5% C15A (457.6 °C), respectively. The overlapping of both registered spectra in the full range of wavenumbers (500–4000 cm^−1^) revealed that, with the exception of slight differences in the absorption bands corresponding to H_2_O and CO_2_, in the case of bands corresponding to the presence of toxic products of PET thermal decomposition (mainly phthalates, benzoic acid homologs and formaldehyde), practically no differences in intensity were observed (Figure 11). There were also no additional bands from the emerging new (toxic) compounds, as observed for, incidentally extremely effective, halogenated flame retardants.

The obtained spectral correlation coefficient was 98.5%. In the case of the second maximum on the G-S curves, corresponding to the maximum evolution of gaseous products at the post-combustion stage of solid char formed after the oxidative degradation process, the value of the spectral correlation coefficient increased up to 99.3%.

### 3.3. Morphology and Crystallinity of Fibers

In order to determine the effect of C15A nanoclay and the conditions of its application on the supermolecular structure of flame-retardant-modified PET fibers, X-ray studies were carried out in both wide (WAXS) and small (SAXS) ranges of diffraction angles. The analysis was carried out for untreated PET fibers processed only in a water bath at 130 °C, and for two variants of the flame-retardant modification i.e., 0.5% (optimal) and 7.5% (maximum) content of C15A nanoclay in the bath.

Using the WAXS method, the basic parameters of the fiber crystal structure, such as mass crystallinity degree and the average size of crystallites, were determined. Moreover, the trend of changes in the structural parameters after the flame-retardant modification of fibers was assessed.

**Figure 12:**
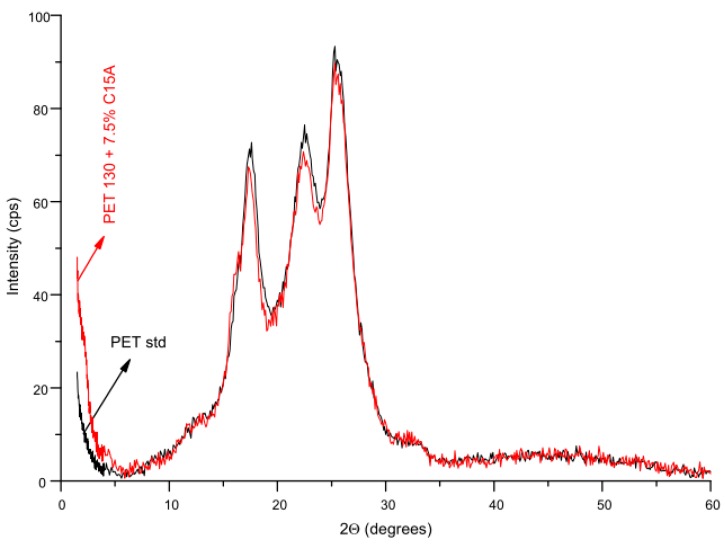
Examples of wide-angle X-ray scattering (WAXS) patterns of fibers subjected to analysis.

In Figure 12, a comparison of WAXS patterns of unmodified PET fibers (PET std) and modified fibers (PET 130 + 7.5% C15A) are presented. For C15A treated fibers, a slight decrease in the intensity of crystalline peaks can be observed, indicating a decrease in the crystallinity of these fibers. PET crystallizes in a triclinic system and according to Daubeney et al. [37], the edges of the unit cell are the single monomeric units of PET homopolymer. PET chains assume an approximately planar configuration. The chain plane is almost parallel to the (100) lattice plane.

**Figure 13:**
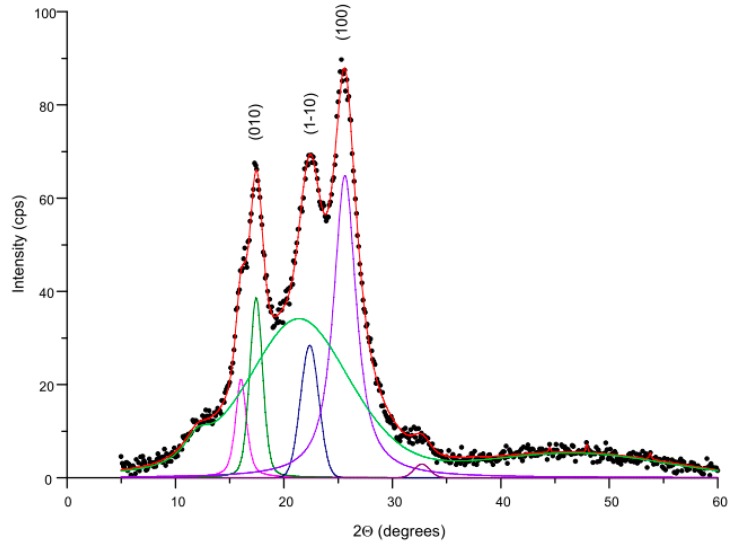
Deconvolution of the exemplary WAXS curve obtained for PET 130 + 7.5% C15A fibers.

Quantitative evaluation of the crystallinity of the fibers represented by the crystallinity index was carried out based on WAXS data. For this purpose, each WAXS curve was deconvoluted into crystalline and amorphous scattering components using WaxsFit profile fitting software [38]. Each peak was modelled using a Gaussian-Cauchy peak shape. The crystallinity index was calculated as a ratio of the area under crystalline peaks to the total area of the scattering curve. Figure 13 is a representative example of a diffraction curve resolved into crystalline and amorphous scattering components using the peak fitting software.

In order to evaluate the variations of crystallite sizes of PET, the Scherrer equation [39] was used. The crystallite sizes were calculated in the direction perpendicular to the (010), (1-10) and (100) planes, i.e., perpendicular to the PET molecular chain axis. The results are presented in Table 2.

Analysis of the results presented in Table 2 indicates that the treatment of fibers in a water bath without the addition of C15A, during 1 h at 130 °C, causes a more than 2% increase in the degree of crystallinity. This was to be expected because during high-temperature processing (coinciding with the temperature range of so-called cold crystallization of PET) the mobility of polymer chains increases, thus further ordering of the structure is possible. It should be noted, however, that the addition of C15A in an amount of only 0.5% (optimal modification variant) caused a more than 4% decrease in the degree of crystallinity. Increasing the amount of nanoclay in the bath to 7.5% caused a further decrease in the degree of fiber crystallinity, but not proportional to the amount of applied modifier. The determined values of the average size of crystallites do not show a specific tendency to change.

**Figure 14:**
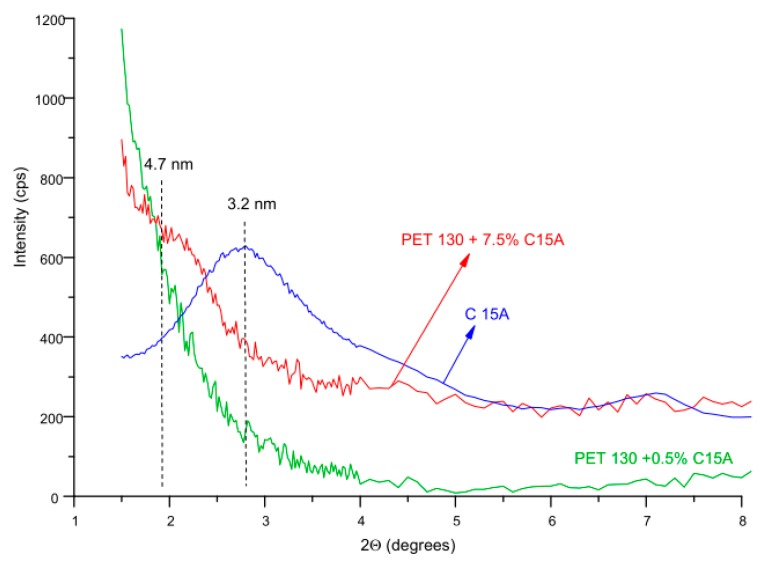
Comparison of the beginning of WAXS curves taken in the angular range corresponding to the characteristic montmorillonite (MMT) peak.

Using the WAXS method, it was additionally possible to assess the degree of dispersion of the aluminosilicate modifier in the fibers. Figure 14 shows the beginning of the diffraction curves for fibers treated with C15A and for pure C15A, covering the angle range corresponding to the characteristic montmorillonite peak, whose angular position is approximately 2.8°. According to Bragg’s law, the position of this peak corresponds to the interlayer spacing of C15A equal to 3.2 nm. In PET 130 + 7.5% C15A samples, this peak shifts towards lower values of the diffraction angle, and the corresponding interlayer spacing of C15A increases to 4.7 nm. This indicates the intercalation of layers, associated with the interaction of montmorillonites with PET chains.

The characteristic montmorillonite peak disappears for the fibers with the optimal flame retardancy (PET + 0.50% of C15A). The WAXS curve of this sample, registered in the analyzed angular range is completely smooth, no local maximum or even inflection point was observed, the location of which would allow to determine the interlayer distance of montmorillonite. The reason for such a diffraction image should not be associated with the finding of the exfoliation of the modifier, but only with too low C15A concentration in the fiber. From the point of view of the WAXS resolution level, the concentration of C15A in the fiber was too low. Demonstrating C15A intercalation itself is already extremely important because it confirms that by using high-temperature treatment of PET fibers under increased pressure, it was possible to introduce a flame retardant in form of C15A to the PET fibers in the solid phase, forming a nanocomposite structure. Our separate work will be devoted to the diffraction and spectroscopic studies of the behavior of intercalated aluminosilicate under the conditions of melting and subsequent combustion of the fibers, and thus explaining the mechanism of flame retardancy.

**Figure 15:**
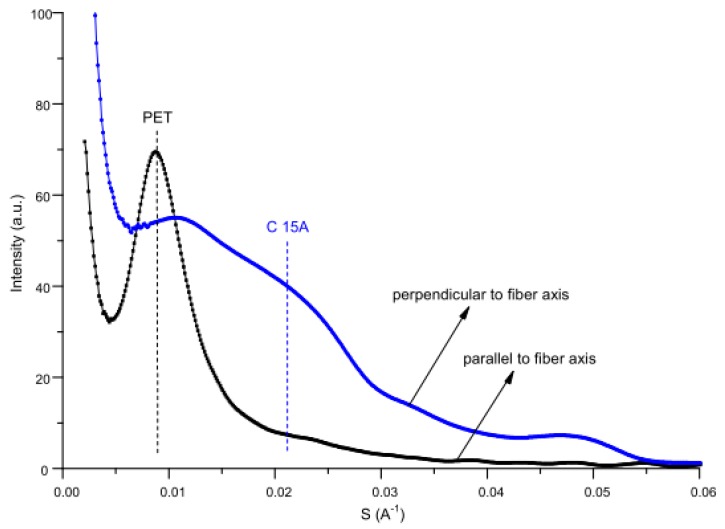
Comparison of small angle X-ray scattering (SAXS) curves taken in the direction parallel and perpendicular to the fiber axis for PET 130 + 7.5% C15A fibers.

In order to understand the distribution of nanoclay in modified fibers, SAXS research was carried out. Investigations performed independently in the direction parallel and perpendicular to the fiber axis indicated another interesting result (Figure 15). The SAXS curve taken in the direction parallel to the fiber axis does not contain the montmorillonite diffraction peak characteristic of C15A, whereas an intense and sharp peak is observed at a relatively small angle. This peak is connected with the lamellar structure of PET. On the other hand, the SAXS curve taken in the direction perpendicular to the fiber axis contains a shoulder which indicates the existence of the montmorillonite peak. These observations reveal that the lamellar structure of PET is perpendicular to the surfaces of montmorillonite layers.

## 4. Conclusions

The following main goals, set by the authors in the course of carrying out the described research, were achieved:

- The addition of C15A clay in the nanopowder form to PET fibers indeed changes the value of the limited oxygen index. The differences in values are not large, but they are undoubtedly significant. The highest LOI value (24.0%) was obtained for PET fibers modified in a bath containing 0.5% C15A to the fiber weight. In this case, the shift (approx. 8.5 °C)of the exothermic peak corresponding to the combustion process of the sample is observed.
- The montmorillonite in form of C15A nanomodifier was successfully introduced into the solid phase PET fiber structure during the modification process in the high-temperature bath, resulting in improved flame retardancy. This was confirmed independently based on the results of LOI tests and TGA tests.
- The use of the TGA-FTIR combined technique has clearly demonstrated no increase in the toxicity of gaseous oxidative degradation products of modified fibers as compared to pure PET fibers, which potentially enables the use the proposed modifier instead of the forbidden halogenated flame retardants.
- The basic parameters of the fiber nanostructure were determined using diffraction methods and their nanocomposite nature was confirmed.

## Figures and Tables

**Table 1:** Limiting oxygen index (LOI) values of studied fibers.

No.	Samples	LOI [%]
1	PET std	21.6
2	PET + 0.10% C15A	23.6
3	PET + 0.25% C15A	23.9
4	PET + 0.50% C15A	24.0
5	PET + 1.00% C15A	23.8
6	PET + 2.50% C15A	20.5
7	PET + 5.00% C15A	20.0
8	PET + 7.50% C15A	19.8

**Table 2:** Crystallinity and dimensions of crystallites obtained by means of WAXS method.

Sample	Crystallinity [%]	Dimensions of Crystallites [nm]		
		D_(010)_	D_(1-10)_	D_(100)_
**PET std**	47.0	5.9	4.6	3.7
PET 130	49.2	5.5	4.2	3.7
PET 130 + 0.5% C15A	45.0	6.3	4.3	4.0
PET 130 + 7.5% C15A	43.9	6.4	4.5	3.7
